# Summer Drinks

**Published:** 1901-05

**Authors:** 


### SUMMER DRINKS.

THE question what to drink in summer is about as easily answered as that on, "What to wear," both depending, of course, upon an array of limitations, foremost of which is individual taste. Scientists are agreed that the ingestion of large quantities of very cold fluids of whatever nature during the mid-summer days is detrimental and therefore injudicious. The smallest quantity of beverage that will quench thirst and give both relief and pleasure is the drink to be preferred. The milder alcoholics, especially the malt liquors, possess some of the necessary qualifications for a good healthy drink, but they also possess a disadvantage in that they contain a variable amount of alcohol which exerts a reactionary influence upon the overheated system. One of the constituents of these malt liquors, which renders them so palatable to many is the carbonic acid with which they are charged, and to this is their thirst quenching properties largely dependent. Another property possessed by these beverages is their stomachic influence, encouraging the flow of the stomach juices and therefore acting as a tonic. Their administration in some of the asthenic conditions of the human system is therefore beneficial and in good practice.

A beverage that will combine the good qualities of the malt liquors exclusive of the alcohol, will come pretty near reaching the goal as an ideal summer drink. Ginger ale is that substitute drink which is pleasant, thirst-quenching, healthful, and therefore meets the requirements of the critical and fastidious. In its manufacture the water entering into its composition is thoroughly aseptisised by heat; the normal oxygen replaced by carbonic acid and the insipid taste of the boiled water replaced by that of syrup of ginger, which is both pleasant, and beneficial. Ginger is an agreeable carminative and stimulant, increasing the stomach and intestinal secretions and promoting peristalsis. It also prevents flatulence and increases the flow of urine. It is therefore an important medicinal agent, and when carefully and methodically mixed with absolutely pure water charged with carbonic acid, furnishes a compound that ought to be more often prescribed by the physician and more frequently imbibed by the laity, especially during the summer months.

Its effect upon febrile conditions is a most happy one. The parched tongue and lips, the nonsecreting membranes of the esophagus and stomach, the sluggish intestine, all cry out for relief, and none comes more quickly than that from a cool draught of sparkling ginger ale.

